# Number of Affected Relatives, Age, Smoking, and Hypertension Prediction Score for Intracranial Aneurysms in Persons With a Family History for Subarachnoid Hemorrhage

**DOI:** 10.1161/STROKEAHA.121.034612

**Published:** 2022-02-11

**Authors:** Charlotte C.M. Zuurbier, Romain Bourcier, Pacôme Constant Dit Beaufils, Richard Redon, Hubert Desal, Anne S.E. Bor, Antti E. Lindgren, Gabriel J.E. Rinkel, Jacoba P. Greving, Ynte M. Ruigrok

**Affiliations:** 1 UMC Utrecht Brain Center, Department of Neurology and Neurosurgery, University Medical Center Utrecht, the Netherlands (C.C.M.Z., G.J.E.R., Y.M.R.).; 2 Department of Diagnostic and Therapeutic Neuroradiology, University Hospital of Nantes, L’institut du thorax, Nantes, Pays de la Loire, FR (R.B., P.C.D.B., R.R., H.D.).; 3 University Hospital Center Nantes, Nantes, Pays de la Loire, France (R.B., P.C.D.B., R.R., H.D.).; 4 Department of Neurology, Rode Kruis Ziekenhuis, Beverwijk, the Netherlands (A.S.E.B.).; 5 Julius Center for Health Sciences and Primary Care, University Medical Center Utrecht, the Netherlands (J.P.G.).; 6 Department of Clinical Radiology and Neurosurgery of NeuroCenter, Kuopio University Hospital, Finland (A.E.L.).; 7 Institute of Clinical Medicine, School of Medicine, Faculty of Health Sciences, University of Eastern Finland, Kuopio, Finland (A.E.L.).

**Keywords:** calibration, hypertension, intracranial aneurysms, prognosis, subarachnoid hemorrhage

## Abstract

Persons with a positive family history of aneurysmal subarachnoid hemorrhage are at increased risk of aneurysmal subarachnoid hemorrhage. Preventive screening for intracranial aneurysms (IAs) in these persons is cost-effective but not very efficient. We aimed to develop and externally validate a model for predicting the probability of an IA at first screening in persons with a positive family history of aneurysmal subarachnoid hemorrhage.

**Methods:**

For model development, we studied results from initial screening for IA in 660 prospectively collected persons with ≥2 affected first-degree relatives screened at the University Medical Center Utrecht. For validation, we studied results from 258 prospectively collected persons screened in the University Hospital of Nantes. We assessed potential predictors of IA presence in multivariable logistic regression analysis. Predictive performance was assessed with the C statistic and a calibration plot and corrected for overfitting.

**Results:**

IA were present in 79 (12%) persons in the development cohort. Predictors were number of affected relatives, age, smoking, and hypertension (NASH). The NASH score had a C statistic of 0.68 (95% CI, 0.62–0.74) and showed good calibration in the development data. Predicted probabilities of an IA at first screening varied from 5% in persons aged 20 to 30 years with two affected relatives, without hypertension who never smoked, up to 36% in persons aged 60 to 70 years with ≥3 affected relatives, who have hypertension and smoke(d). In the external validation data IA were present in 67 (26%) persons, the model had a C statistic of 0.64 (95% CI, 0.57–0.71) and slightly underestimated IAs risk.

**Conclusions:**

For persons with ≥2 affected first-degree relatives, the NASH score improves current predictions and provides risk estimates for an IA at first screening between 5% and 36% based on 4 easily retrievable predictors. With the information such persons can now make a better informed decision about whether or not to undergo preventive screening.

Persons with a positive family history for aneurysmal subarachnoid hemorrhage (aSAH) have an increased risk of aSAH. According to the number of affected relatives, the lifetime risk of aSAH can be as high as 25%.^1^ Early diagnosis of unruptured intracranial aneurysms (IAs) can influence clinical management and prognosis, as timely intervention might prevent aSAH. In persons with 2 or more affected first-degree relatives preventive screening for IAs is cost-effective when this is repeated every 5 to 7 years between 20 and 70 to 80 years of age.^2,3^

During screening in persons with a positive family history of aSAH an IA is found at initial screening in only 10%.^4,5^ Early risk stratification of persons with IAs may help to identify persons at high or low risk of IAs, and thereby improve efficiency of screening.

Several prognostic factors increase the likelihood of having an IA in the general population. These include older age, female sex, cigarette smoking, history of hypertension, history of aSAH, and positive family history for aSAH.^5–7^ In persons with familial aSAH screened for IAs, all these factors were also found to be associated with an increased risk of having an IA.^4,8–11^

We aimed to develop and externally validate a prediction model for predicting the probability of an IA at first screening in persons with a positive family history of aSAH.

## Methods

### Study Population

The data that support the findings of this study are available from the corresponding author upon reasonable request. For the development of the model we used a prospectively collected cohort of 660 persons, with 2 or more first-degree relatives who had aSAH, or persons with one first-degree relative with aSAH and one or more first-degree relative with an unruptured IA, who were screened for IA at the University Medical Center Utrecht, the Netherlands. Screening for IA at this center started in April 1993, and we retrieved all available information from April 1993 up to April 2020. All persons with aSAH who were admitted or persons with an IA who visited the outpatient clinic at the University Medical Center Utrecht were routinely asked for details about their family history. If aSAH occurred in their relatives, we informed them that their relatives were welcome to visit the outpatient clinic to be informed about screening for IA. Persons were also referred for screening by general practitioners, neurologists, or neurosurgeons.

We included all persons with 2 or more first-degree relatives (parents, siblings, or children) who had had a definite or probable aSAH. We also included persons with one first-degree relative with aSAH and another first-degree relative with an unruptured IA proven by CT-angiography, magnetic resonance angiography, or digital subtraction angiography. Definite aSAH was defined as an abrupt onset of severe headache or loss of consciousness with or without focal neurological signs, the presence of subarachnoid blood on head CT compatible with a ruptured IA and an IA on CT-angiography, magnetic resonance angiography, or digital subtraction angiography. Probable aSAH was defined as an episode suspected to be aSAH in a person younger than 70 years, such as stroke with a second ictus within 4 weeks followed by death.^12^ The standard screening modality was magnetic resonance angiography, and in case of contraindications, screening was performed by CT-angiography instead. Screening was usually performed from the age of 18 years until the age of ≈70 years, with the precise cutoff depending on the state of health of the screenees. Performed screening reflects clinical practice, and was not according to a study protocol. We excluded persons screened for IAs because of autosomal dominant polycystic kidney disease. The 660 persons included were selected from a total cohort of 935 persons screened between April 1993 up to April 2020. The main reasons for not including the remaining 275 persons were (1) relatives were not related in the first degree and did not have definite or probable aSAH (n=207); (2) only one affected first-degree relative (n=59); autosomal dominant polycystic kidney disease (n=9). The outcome of interest was the presence of an IA at first screening.

### Model Development

We obtained information about candidate predictors preselected based on the literature, which included age, sex, smoking, history of hypertension, history of previous aSAH, and number of affected family members with aSAH and IAs.^4–9^ Smoking was defined as former or current smoking, and hypertension as a history of hypertension or use of antihypertensive drugs. The number of affected family members with aSAH or IAs was categorized into 2 affected relatives versus 3 or more affected relatives.

### External Validation

For external validation of the model we used the Understanding the Pathophysiology of Intracranial Aneurysm (ICAN) prospective familial IA cohort consisting of 265 persons screened for IA because of familial aSAH in France between December 2012 and April 2019.^13^

### Statistical Analysis

The proportion of missing data within the development data was zero for most candidate predictors, except for smoking (34%) and hypertension (37%). Missing data were imputed with multiple imputation, creating 10 imputed data sets. In the validation cohort, data were missing on hypertension for 7 cases (3%) and these 7 cases were excluded from the analysis. Restricted cubic splines were used to assess whether continuous predictors (age) could be analyzed as linear term or needed transformation. Age showed a linear association with the outcome. We performed multivariable logistic regression analysis to study the association between candidate predictors and the presence of an IA at first screening. We studied this association in all 10 imputed data sets. All potential predictors were considered for inclusion in the model regardless of their association in the univariable analysis, and the model was simplified by performing backward selection based on Akaike Information Criterion.^14^ The interaction between age and number of affected family members was included in the model, as with older age more affected family members can expected to be found. Because prognostic models derived from multivariable regression analysis can be optimistic and thereby overestimate predictions when applied to a new cohort of persons,^15,16^ we internally validated the model with bootstrapping techniques. A shrinkage factor was estimated from the bootstrap procedure and regression coefficients were multiplied by this shrinkage factor to correct for overfitting. The regression coefficients in each imputation data set were pooled with Rubin rules.^17^ We examined the performance of the final prediction model by determining its discrimination and calibration. Discrimination refers to what extent the model distinguishes between individuals with and without an IA and was assessed with the concordance (C) statistic, which was corrected for over optimism. We pooled the C statistics of each multiply imputed data set with Rubin rules. Calibration refers to the agreement between observed and predicted risk and was studied with a calibration plot. To facilitate practical application of the model, we used the regression coefficients of the predictors in the final model, to allocate points to each predictor to generate a risk score. We translated the regression model into a score chart by dividing all regression coefficients by the smallest coefficient and subsequently rounded them to the nearest integer. The score chart is accompanied by a figure and table displaying estimated IA risks at first screening.

For external validation, we applied the original regression equation to the validation data from the French cohort and calculated the predicted probability of finding IAs at first screening for each person. We assessed model performance with the C statistic and calibration plots. Results are reported in accordance with the Transparent Reporting of a multivariable prediction model for Individual Prognosis or Diagnosis statement.^18^ The study was approved by the Medical Ethics Committee of the University Medical Center Utrecht, the Netherlands and all subjects provided oral informed consent before screening.

## Results

The baseline characteristics of the persons of the development and validation cohorts are presented in Table 1. Among 660 persons included in the development cohort, 79 (12%) had an IA at first screening. Of these persons, 26 (33%) persons had multiple IAs (for all IA characteristics, see Table S1). In the validation cohort an IA was found in 67 of 258 persons (26%) of whom 21 (31%) persons had multiple IAs. Persons in the validation cohort were slightly older (mean age 40±14 years versus 48±15 years), were more often current or past smokers (54% versus 68%), and more often had 3 or more affected family members than persons in the development cohort (48% versus 66%).

**Table 1.**
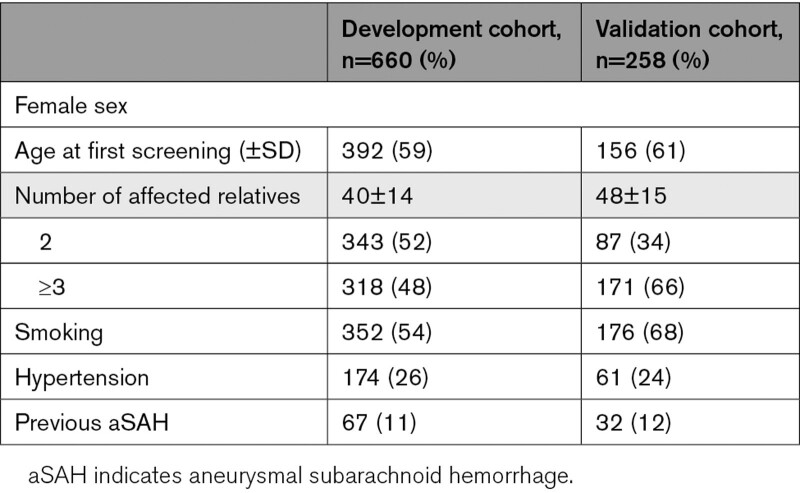
Baseline Characteristics of Persons of the Development and External Validation Cohort

The results of the multivariable logistic regression analysis are presented in Table 2. The following predictors were identified: number of affected family members ≥3, older age, smoking, hypertension, and the interaction between age and number of affected family members ≥3 (number of affected relatives, age, smoking, hypertension [NASH]).

**Table 2.**
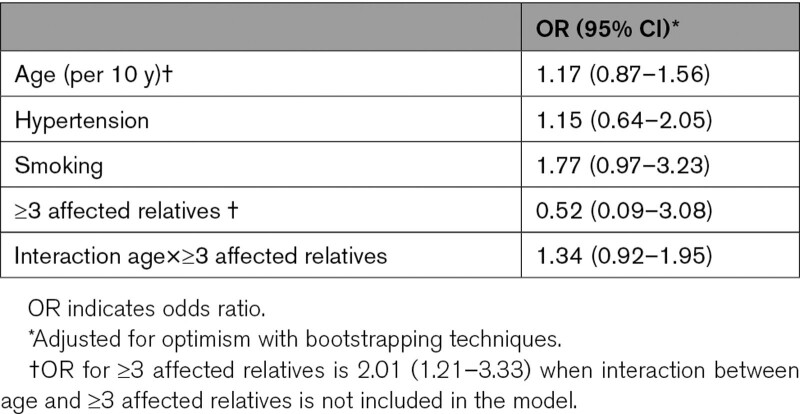
Multivariable Ratios for Risk of Intracranial Aneurysm From the Final Model After Shrinkage

**Figure 1.**
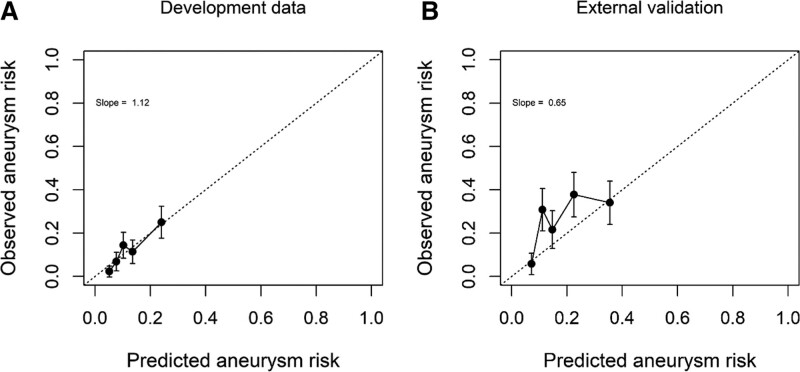
**Calibration plot. A**, In the development cohort (**B**) in the validation cohort.

After shrinkage, the model had a C statistic of 0.68 (95% CI, 0.62–0.74). The calibration plot showed good correspondence between predicted and observed risk (Figure 1). The original regression equation is provided in Table S2.

**Figure 2.**
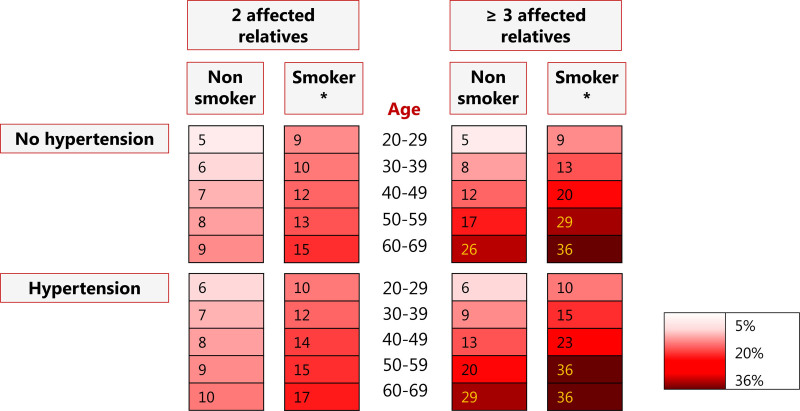
**Prediction chart with absolute probabilities (%) of an intracranial aneurysm at first screening.** *Former or current smoker.

We translated regression coefficients into a score chart presented in Table S3. Our NASH score can be used in combination with Table S4 and Figure S1 to obtain predicted probabilities for individual persons. Figure 2 shows a risk chart with estimated probabilities of finding an IA at first screening according to age, smoking status, hypertension status, and number of affected family members. The probability of finding an IA ranged from 5% in persons aged 20 to 30 years with 2 affected relatives, without hypertension who never smoked, up to 36% in persons aged 60 to 70 years with 3 or more affected relatives, who have hypertension, and smoke or have smoked in the past.

### External Validation

External validation of the NASH model showed a C statistic of 0.64 (95% CI, 0.57–0.71). The calibration plot shows that the likelihood of finding an IA increased along the range of predicted probabilities. The prediction score slightly underestimated the probability of finding an IA, in particular in the middle risk quintile. Overall, observed risks were within the range of expected risks with moderate calibration.

## Discussion

We developed the NASH score that predicts the risk of IAs at first screening in persons with a positive family history of aSAH. Based on the number of affected relatives, age, smoking, and hypertension, the risk of IA can vary from 5% in persons aged 20 to 30 years with 2 affected relatives, who have no hypertension and never smoked, to 36% in persons aged 60 to 70 years with 3 or more affected relatives, who have hypertension, and smoke or have smoked in the past.

We found that sex and previous aSAH had no added value for the prediction of an IA at first screening when other risk factors were taken into account The limited role of sex as a predictor of IAs in persons with a positive family history of aSAH may be caused by a less dominant role of sex in these persons compared with persons with a negative family history. IAs are more prevalent in woman than in men in the general population,^6^ but in studies with familial patients the difference between women and men with IAs is less profound.^19,20^ The lack of added value of a previous aSAH in the risk of finding an IA at screening may be explained by the fact that aSAH patients with a positive family history of aSAH were advised repeated screening for de novo IAs 5 years after having their aSAH. Consequently, 5 years might have been too short to develop a de novo IA.

In our study, we found an OR for hypertension of 1.15, with upper range of 2.05 of the 95% CI when other risk factors were taken into account. This was lower than expected as hypertension has been identified as a stronger risk factor for unruptured IA with odds ratios ranging from 2.2 to 2.9 in previous studies.^21^ Moreover, in a study on risk factors for unruptured IA specifically in persons with a positive family history of aSAH a comparable odds ratio for hypertension of 1.9 (95% CI, 1.0–3.7) was found.^10^ These studies used the same definition for hypertension which definition was also used in our current study. However, data on the precise risk of hypertension are inconsistent as a more recent study on risk factors for unruptured IAs an association with hypertension could not be established.^22^ More data on the role of hypertension in the development of unruptured IAs in both persons with and without a positive family history for aSAH are needed using a large prospective cohort and taking into account other risk factors associated with an increased risk of IA.

Although the observed and predicted IA risk corresponded accurate in the development data, the predicted IA risk was slightly underestimated in the external validation data. This is likely due to differences between the development and validation cohort in terms of included persons. In the validation cohort, more persons had an IA (26%) than in the development cohort (12%). This may have resulted in an underestimated risk of finding an IA when the prediction model was applied in the validation cohort. In addition, selection of persons at high risk in the validation cohort may also have altered predictor-outcome associations. As a consequence, the ability of the model to distinguish between individuals with and without an IA may have decreased.

Strengths of our study include the prospectively collected data of the development and validation cohort. Moreover, the data used for development of the model encompassed the entire period that our center has offered screening to persons with a positive family history of aSAH and included a large sample size, which enabled us to study a broad range of prognostic factors. Another strength is the external validation using data from another center based in another country. Our study also has limitations that need to be considered. First, despite the prospective data collection, still some data on smoking and hypertension were missing in our development cohort. However, multiple imputation was used to predict missing values with information from all potential predictors and outcome. Thus, we were able to include all persons in our model, which resulted in a prediction rule with high precision. Second, although the current model provides risk estimates for IA development at first screening, we have no individualized data on risks of IA at follow-up screening as in this study we only included data at first screening and did not include data at follow-up screening. In general, the risk of finding a new aneurysm 5 years after a negative screen is around 5-7%,^4^ but the influence of risk factors on this proportion is unknown. Finally, we did not study persons screened with only one affected first-degree relative and, therefore, our results cannot be extrapolated to persons screened for IA who have only one affected relative. For persons with only one affected relative as a group, screening twice, at age 40 and 55, is cost-effective,^23^ but if and how risk factors affect this strategy is yet unknown.

Our risk prediction chart based on easily available patient characteristics predicts the probability of finding an IA at first screening in persons with 2 or more affected first-degree relatives. Based on the risk estimates from the prediction model, persons with a positive family history of aSAH can now make a better informed decision about whether or not to undergo preventive screening. Future studies should assess individualized risk prediction of IA during follow-up screening and develop a decision model to define the optimal screening strategies in persons based on their individualized risk of IA development. Persons with a high risk of IAs can have intensified screening, while in persons with a lower risk screening may be reduced. Future studies should also assess individualized risk prediction of IA for persons with only one affected first-degree relative.

## Article Information

### Acknowledgments

Dr Zuurbier, Dr Ruigrok, and Dr Rinkel contributed to the study design. Dr Zuurbier performed the data analysis under the supervision of Drs Ruigrok, Greving, and Rinkel. Dr Zuurbier wrote the first draft of the article and all authors revised the article critically and approved the final version.

### Sources of Funding

This study was supported by the Netherlands Cardiovascular Research Initiative: An initiative with support of the Dutch Heart Foundation, CVON2015-08 ERASE. This project has received funding from the European Research Council (ERC) under the European Union’s Horizon 2020 research and innovation programme (PRYSM, grant agreement No. 852173). Dr Zuurbier was supported by the Remmert Adriaan Laan Foundation. Dr Zuurbier reports a grant from Remmert Adriaan Laan Foundation, during the conduct of the study. We are grateful to the Clinical Investigation Center (INSERM CIC1413) for its assistance in managing the ICAN biobanks. Dr Redon was supported by the French Regional Council of Pays-de-la-Loire (VaCaRMe program) and the Agence Nationale de la Recherche (ANR-15-CE17-0008-01 to G.L), Drs Desal and Bourcier were supported by the French Ministry of Health (Clinical trial URL: https://www.clinicaltrials.gov; Unique identifier: NCT02848495 to Dr Desal), the Genavie Foundation, the Société Française de Radiologie and the Société française de Neuroradiologie. The funding organizations were not involved in the design and conduct of the study; the collection, management, analysis, and interpretation of the data; the preparation, review, or approval of the article; and decision to submit the article for publication

### Disclosures

None.

### Supplemental Material

Tables S1–S4

Figure S1

## Supplementary Material



## References

[1] Bor AS Rinkel GJ Adami J Koffijberg H Ekbom A Buskens E Blomqvist P Granath F. Risk of subarachnoid haemorrhage according to number of affected relatives: a population based case-control study. Brain. 2008;131(pt 10):2662–2665. doi: 10.1093/brain/awn187 18819992 10.1093/brain/awn187

[2] Bor AS Koffijberg H Wermer MJ Rinkel GJ. Optimal screening strategy for familial intracranial aneurysms: a cost-effectiveness analysis. Neurology. 2010;74:1671–1679. doi: 10.1212/WNL.0b013e3181e04297 20498435 10.1212/WNL.0b013e3181e04297

[3] Takao H Nojo T Ohtomo K. Screening for familial intracranial aneurysms: decision and cost-effectiveness analysis. Acad Radiol. 2008;15:462–471. doi: 10.1016/j.acra.2007.11.007 18342771 10.1016/j.acra.2007.11.007

[4] Bor AS Rinkel GJ van Norden J Wermer MJ. Long-term, serial screening for intracranial aneurysms in individuals with a family history of aneurysmal subarachnoid haemorrhage: a cohort study. Lancet Neurol. 2014;13:385–392. doi: 10.1016/S1474-4422(14)70021-3 24618352 10.1016/S1474-4422(14)70021-3

[5] Magnetic Resonance Angiography in Relatives of Patients with Subarachnoid Hemorrhage Study G. Risks and benefits of screening for intracranial aneurysms in first-degree relatives of patients with sporadic subarachnoid hemorrhage. N Engl J Med. 1999;341:1344–1350. doi: 10.1056/NEJM199910283411803 10536126 10.1056/NEJM199910283411803

[6] Vlak MH Algra A Brandenburg R Rinkel GJ. Prevalence of unruptured intracranial aneurysms, with emphasis on sex, age, comorbidity, country, and time period: a systematic review and meta-analysis. Lancet Neurol. 2011;10:626–636. doi: 10.1016/S1474-4422(11)70109-0 21641282 10.1016/S1474-4422(11)70109-0

[7] Brown RD Jr Broderick JP. Unruptured intracranial aneurysms: epidemiology, natural history, management options, and familial screening. Lancet Neurol. 2014;13:393–404. doi: 10.1016/S1474-4422(14)70015-8 24646873 10.1016/S1474-4422(14)70015-8

[8] Brown RD Jr Huston J Hornung R Foroud T Kallmes DF Kleindorfer D Meissner I Woo D Sauerbeck L Broderick J. Screening for brain aneurysm in the Familial Intracranial Aneurysm study: frequency and predictors of lesion detection. J Neurosurg. 2008;108:1132–1138. doi: 10.3171/JNS/2008/108/6/1132 18518716 10.3171/JNS/2008/108/6/1132 PMC4190025

[9] Raaymakers TW. Aneurysms in relatives of patients with subarachnoid hemorrhage: frequency and risk factors. MARS Study Group. Magnetic Resonance Angiography in Relatives of patients with Subarachnoid hemorrhage. Neurology. 1999;53:982–988. doi: 10.1212/wnl.53.5.982 10496256 10.1212/wnl.53.5.982

[10] Rasing I Nieuwkamp DJ Algra A Rinkel GJ. Additional risk of hypertension and smoking for aneurysms in people with a family history of subarachnoid haemorrhage. J Neurol Neurosurg Psychiatry. 2012;83:541–542. doi: 10.1136/jnnp-2011-301147 22423116 10.1136/jnnp-2011-301147

[11] Connolly ES Jr Choudhri TF Mack WJ Mocco J Spinks TJ Slosberg J Lin T Huang J Solomon RA. Influence of smoking, hypertension, and sex on the phenotypic expression of familial intracranial aneurysms in siblings. Neurosurgery. 2001;48:64–8; discussion 68. doi: 10.1097/00006123-200101000-00011 11152362 10.1097/00006123-200101000-00011

[12] Bromberg JE Rinkel GJ Algra A Greebe P Beldman T van Gijn J. Validation of family history in subarachnoid hemorrhage. Stroke. 1996;27:630–632. doi: 10.1161/01.str.27.4.630 8614920 10.1161/01.str.27.4.630

[13] Bourcier R Chatel S Bourcereau E Jouan S Marec HL Daumas-Duport B Sevin-Allouet M Guillon B Roualdes V Riem T; ICAN Investigators. Understanding the Pathophysiology of Intracranial Aneurysm: The ICAN Project. Neurosurgery. 2017;80:621–626. doi: 10.1093/neuros/nyw135 28362927 10.1093/neuros/nyw135

[14] Royston P Moons KG Altman DG Vergouwe Y. Prognosis and prognostic research: developing a prognostic model. BMJ. 2009;338:b604. doi: 10.1136/bmj.b604 19336487 10.1136/bmj.b604

[15] Harrell FE Jr Lee KL Mark DB. Multivariable prognostic models: issues in developing models, evaluating assumptions and adequacy, and measuring and reducing errors. Stat Med. 1996;15:361–387. doi: 10.1002/(SICI)1097-0258(19960229)15:4<361::AID-SIM168>3.0.CO;2-4 8668867 10.1002/(SICI)1097-0258(19960229)15:4<361::AID-SIM168>3.0.CO;2-4

[16] Altman DG Vergouwe Y Royston P Moons KG. Prognosis and prognostic research: validating a prognostic model. BMJ. 2009;338:b605. doi: 10.1136/bmj.b605 19477892 10.1136/bmj.b605

[17] van Buuren S Groothuis-Oudshoorn K. Mice: multivariate imputation by chained equations in R. J Stat Sofw. 2011;45:1–67. doi: 10.18637/jss.v045.i03

[18] Collins GS Reitsma JB Altman DG Moons KG. Transparent reporting of a multivariable prediction model for individual prognosis or diagnosis (TRIPOD): the TRIPOD statement. BMJ. 2015;350:g7594. doi: 10.1136/bmj.g7594 25569120 10.1136/bmj.g7594

[19] Wills S Ronkainen A van der Voet M Kuivaniemi H Helin K Leinonen E Frösen J Niemela M Jääskeläinen J Hernesniemi J. Familial intracranial aneurysms: an analysis of 346 multiplex Finnish families. Stroke. 2003;34:1370–1374. doi: 10.1161/01.STR.0000072822.35605.8B 12750547 10.1161/01.STR.0000072822.35605.8B

[20] Ruigrok YM Rinkel GJ Algra A Raaymakers TW Van Gijn J. Characteristics of intracranial aneurysms in patients with familial subarachnoid hemorrhage. Neurology. 2004;62:891–894. doi: 10.1212/01.wnl.0000115104.19787.8e 15037688 10.1212/01.wnl.0000115104.19787.8e

[21] Kang HG Kim BJ Lee J Kim MJ Kang DW Kim JS Kwon SU. Risk factors associated with the presence of unruptured intracranial aneurysms. Stroke. 2015;46:3093–3098. doi: 10.1161/STROKEAHA.115.011351 26451026 10.1161/STROKEAHA.115.011351

[22] Müller TB Vik A Romundstad PR Sandvei MS. Risk factors for unruptured intracranial aneurysms and subarachnoid hemorrhage in a prospective population-based study. Stroke. 2019;50:2952–2955. doi: 10.1161/STROKEAHA.119.025951 31370767 10.1161/STROKEAHA.119.025951

[23] Hopmans EM Ruigrok YM Bor AS Rinkel GJ Koffijberg H. A cost-effectiveness analysis of screening for intracranial aneurysms in persons with one first-degree relative with subarachnoid haemorrhage. Eur Stroke J. 2016;1:320–329. doi: 10.1177/2396987316674862 31008294 10.1177/2396987316674862 PMC6301253

