# Supplementary material for: Number of Affected Relatives, Age, Smoking, and Hypertension Prediction Score for Intracranial Aneurysms in Persons With a Family History for Subarachnoid Hemorrhage
Source: Stroke. 2022 Feb 11;53(5):1645–50. doi: 10.1161/STROKEAHA.121.034612 (PMC9022690; doi:10.1161/STROKEAHA.121.034612)

SUPPLEMENTAL MATERIAL

Supplemental Table I: Characteristics of intracranial aneurysms identified in the development and the validation cohort

	**Development cohort n= 114 (%)**	**Validation cohort* n = 99 (%)**
Location		
- Internal carotid artery	50 (44)	36 (36)
- Middle cerebral artery	38 (33)	41 (41)
- Anterior communicating artery	20 (18)	14 (14)
- Posterior circulation	6 (5)	4 (4)
Size		
- <2 mm	32 (28)	13 (13)
- 2-5 mm	60 (53)	65 (65)
- > 5mm	22 (19)	11 (11)

* data on location missing in 4 persons in validation cohort and data on size missing in 10 persons in validation cohort

Supplemental Table II: Number of affected relatives in the development cohort and the validation cohort

	**Development cohort n=660 (%)**	**Validation cohort n=258 (%)**
Number of affected relatives		
- 2	343 (52)	87 (34)
- 3	152 (23)	91 (35)
- 4	55 (8)	71 (28)
- 5	44 (7)	9 (4)
- 6	32 (5)	0
- 7	7 (1)	0
- 8	1 (0)	0
- 9	27 (4)	0

Supplemental Table III: Regression equations of multivariable models

Regression equation model based on person characteristics
-3.324946 -0.64194*≥3Affectedrelatives+0.01532*Age+0.13517*Hypertension+0.56427* Smoking+0.02924*Interaction Age and ≥3Affected relatives

Supplemental Figure I: Predicted probability of an intracranial aneurysm at first screening based on the NASH prediction score (graphic display of Table IV)

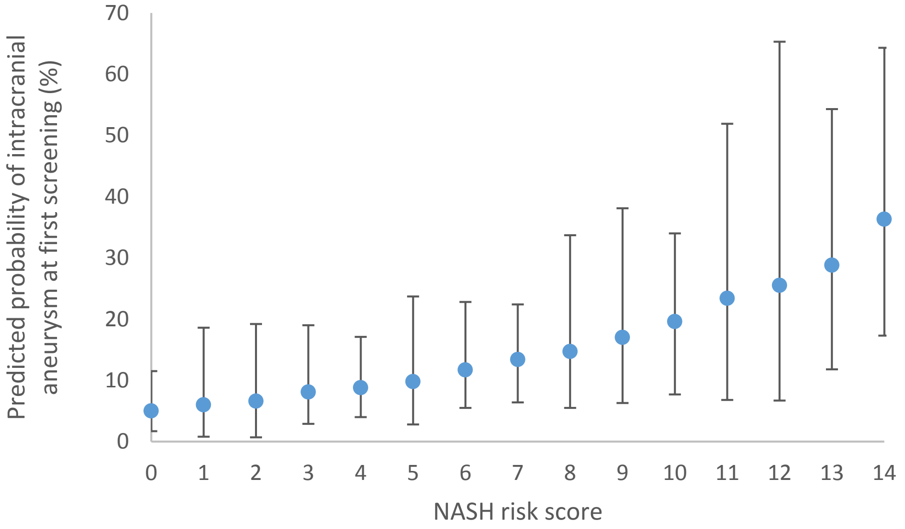

Supplement: Supplementary file 1 [file str-53-1645-s001.pdf]
